# Molecular tumor analysis and liquid biopsy: a feasibility investigation analyzing circulating tumor DNA in patients with central nervous system lymphomas

**DOI:** 10.1186/s12885-019-5394-x

**Published:** 2019-03-01

**Authors:** Anne-Katrin Hickmann, Maximilian Frick, Dirk Hadaschik, Florian Battke, Markus Bittl, Oliver Ganslandt, Saskia Biskup, Dennis Döcker

**Affiliations:** 1 Department of Neurosurgery, Kantonsspital St. Gallen, Rorschacherstrasse 95, 9600 St. Gallen, Switzerland; 2 Neurosurgical Department, Klinikum Stuttgart, Stuttgart, Germany; 3 Center for Genomics and Transcriptomics (CeGaT) GmbH, Tübingen, Germany; 4 Hertie Institute for Clinical Brain Research, University of Tübingen, Tübingen, Germany; 5 Outpatient Clinic for Human Genetics, Tübingen, Germany

**Keywords:** Cerebrospinal fluid, Circulating DNA, CNS-lymphoma, Liquid biopsy, Personalized medicine, Targeted therapies

## Abstract

**Background:**

Central nervous system lymphomas (CNSL) is a devastating disease. Currently, a confirmatory biopsy is required prior to treatment.

**Objective:**

Our investigation aims to prove the feasibility of a minimally-invasive diagnostic approach for the molecular characterization of CNSL.

**Methods:**

Tissue biopsies from 6 patients with suspected CNSL were analyzed using a 649gene next-generation sequencing (NGS) tumor panel (tumor vs. reference tissue (EDTA-blood)). The individual somatic mutation pattern was used as a basis for the digital PCR analyzing circulating tumor DNA (ctDNA) from plasma and cerebrospinal fluid (CSF) samples, identifying one selected tumor mutation during this first step of the feasibility investigation.

**Results:**

NGS-analysis of biopsy tissue revealed a specific somatic mutation pattern in all confirmed lymphoma samples (*n* = 5, NGS-sensitivity 100%) and none in the sample identified as normal brain tissue (NGS-specificity 100%). cfDNA-extraction was dependent on the extraction-kit used and feasible in 3 samples, in all of which somatic mutations were detectable (100%). Analysis of CSF-derived cfDNA was superior to plasma-derived cfDNA and routine microscopic analysis (lymphoma cells: *n* = 2, 40%). One patient showed a divergent molecular pattern, typical of Burkitt-Lymphoma (HIV+, serologic evidence of EBV-infection). Lumbar puncture was tolerated without complications, whereas biopsy caused 3 hemorrhages.

**Conclusions:**

Our investigation provides evidence that analysis of cfDNA in central nervous system tumors is feasible using the described protocol. Molecular characterization of CNSL could be achieved by analysis of CSF-derived cfDNA. Knowledge of a tumor’s specific mutation pattern may allow initiation of targeted therapies, treatment surveillance and could lead to minimally-invasive diagnostics in the future.

**Electronic supplementary material:**

The online version of this article (10.1186/s12885-019-5394-x) contains supplementary material, which is available to authorized users.

## Background

Central nervous system lymphoma (CNSL) is a rare entity among intracranial neoplasms, comprising only 4% of all intracranial tumors [1]. Thus its verification is essential prior to initiation of an aggressive treatment varying from the one for its differentials (e.g. metastasis, glioma) [1, 2]. Needle biopsy remains the gold standard with a diagnostic accuracy of 73–97% and a considerable procedural morbidity (≤16.1%) and mortality (≤3.9%), requiring re-operations in selected cases [3].

Lately, circulating free DNA (cfDNA) has been increasingly investigated for its utilization in tumor-diagnosis and surveillance [4–9], thereby the tumor-specific fraction of cfDNA is referred to as circulating tumor DNA (ctDNA). In primary brain tumors, several molecular markers and circulating proteins have been identified using peripheral blood samples [10–12]. Nonetheless, the plasma may not be optimal for detection of ctDNA from CNS-tumors due to the blood brain barrier [10, 13].

We investigated the feasibility of molecular characterization of CNSL based on tissue samples applying next-generation sequencing (NGS) and detection of specific mutations in the periphery analyzing ctDNA from plasma and cerebrospinal fluid (CSF), which could aid in treatment surveillance and compilation of personalized treatment plans.

## Methods

### Patient selection

Patients with suspected CNSL, scheduled for a biopsy at our neurosurgical department, were evaluated for eligibility prior to initiation of the mandatory diagnostic workup. Inclusion criteria were 1) suspected CNSL based on clinical presentation and cranial imaging (CT and/or MRI), 2) no contraindications to surgery, 3) age ≥ 18 years 4) written informed consent by patient or legally competent next of kin.

### Tissue biopsy and postoperative care

Tissue biopsies were taken from patients with suspected CNSL according to local standard on surgeon’s preference (*n* = 2 open biopsy, *n* = 4 stereotactic). For a stereotactic biopsy, all patients received a preoperative contrast-enhanced cranial CT with the Leksell frame (Elekta AB, Stockholm, Sweden) in place. The CT was then transferred to a BrainLab working station (BrainLab AG, München, Germany) to plan the optimal trajectory (patients #1,2,3,5) while the patient was transferred to the OR and prepped for surgery. Per patient 8–12 cylinders of tumor tissue were taken. Open biopsy was performed navigation-assisted (BrainLab) through a 2 × 3 cm craniotomy in patient #4 because of the possible differential diagnosis of a glioblastoma with the option of tumor resection during the same surgery (lymphoma confirmed during intraoperative frozen section analysis, termination of surgery) and in patient #6 because of a superficial lesion affecting cortex (hypervascularity) and meninges (thickened, contrast-enhancing). An approximately 1cm^3^ specimen was taken per patient.

According to local standards, all patients undergoing stereotactic biopsy were postoperatively monitored for 2–4 h in the recovery room and then transferred back to their previous ward, if neurologically stable. Otherwise further imaging (CT) and/or transfer to an intermediate care unit (IMC)/intensive care unit (ICU), as appropriate, was initiated. Cranial CT scans were performed on postoperative day 1.

Patients undergoing open biopsy were monitored in the ICU for the first 24 h postoperatively and then transferred back to their previous ward after having received a routine postoperative cranial imaging (CT / MRI) without signs of complication. If neurological deficits became apparent during monitoring in the ICU prompt imaging was initiated.

Once tissue biopsy was completed, all patients received high dose steroids (12 mg/day, single dose in the morning). The anesthesiologist gave the first dose intra-operatively, irrespective of daytime. After 2–4 days in the neurosurgical unit, patients were transferred to hematology/oncology for further treatment once histopathology reports returned.

### Assessment of somatic variants in tumor/control tissue

The routine pathological evaluation was performed on the biopsied tissue and a small sample was sent for genetic analysis (requirements: 1 μg input DNA). EDTA blood, taken during routine preoperative workup, served as reference tissue.

Genomic DNA from tumor and reference tissue was extracted using the QIAamp DNA Blood Mini Kit (Qiagen GmbH, Hilden/Germany). Somatic variants were detected by next-generation sequencing (TUM01 somatic-tumor-panel (649 genes, 28 specific translocations, CeGaT, Tübingen/Germany, complete gene list see Additional file 1: section 1).

### cfDNA analyses

As part of the routine workup for CNLS, all patients underwent a lumbar puncture to rule out infectious or autoimmunologic disease and for pathologic as well as cytologic evaluation. 1-4 ml of remaining CSF were collected in cfDNA-BCT®-Tubes (Streck, Omaha, NE/USA) for dPCR. At the time of lumbar puncture a second blood sample was drawn (cfDNA-BCT®-Tubes, Streck, Omaha, NE/USA) to ensure comparability between plasma and CSF, since tumor tissue does not break down continuously. On the same day, the cellular fraction was removed by centrifugation (10 min at 1900 g and 4 °C). A subsequent, second centrifugation was performed at 16,000 g and 4 °C to remove any remaining cellular debris. The isolated plasma and CSF samples were stored at − 80 °C till further processing. The extraction of cfDNA from plasma and CSF was first performed using the Polymer Mediated Enrichment (PME) free circulating DNA Extraction Kit (Analytik Jena, Germany), but was replaced with the QIAamp Circulating Nucleic Acid Kit (Qiagen GmbH, Hilden, Germany), due to unsatisfactory results and based on general experience in our laboratory. Isolated cfDNA was stored at − 20 °C until analysis.

**Fig. 1:**
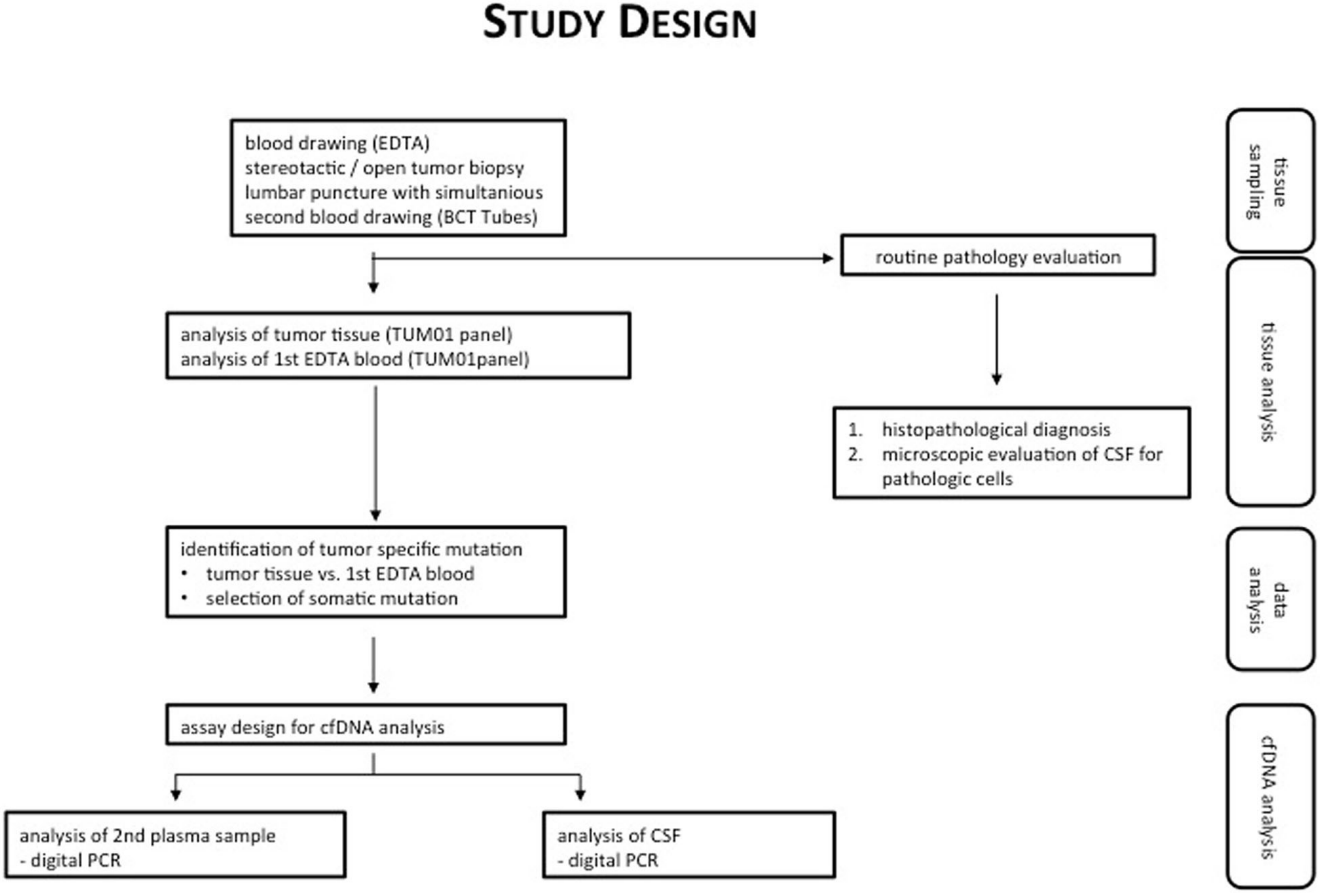
Study design

From the detected somatic mutations within the tumor tissue (NGS), one *missense* variant was selected (the one with the highest mutant allele frequency (MAF), phylogenetically older, possible driver mutation) followed by assaying its presence in plasma- and CSF-cfDNA using individually designed duplex-TaqMan assays (ThermoFisher Scientific, Waltham, MA, USA) and digital PCR (dPCR) (BioRad QX200 Bio-Rad Laboratories, Hercules, CA/USA). Specificity of the primers’ and probes’ sequences was manually checked by the authors (MF, DD) with the Basic Local Alignment Search Tool (BLAST, NCBI, with both GRCh37/38 reference) (for sequences see Additional file 1: section 2). dPCR was chosen, because it generally allows the detection of very low allele frequencies down to < 0.01%, depending on input cfDNA amount. Further details about dPCR, including statistical interpretation, are outlined in the Additional files (Additional file 1: section 3, Additional file 2: Figure S1, Additional file 3: Figure S2). Figure 1 outlines the study design.

### Statistics

Based on the results of this investigation we performed a sample size analysis to guide future investigations evaluating concentration of cfDNA and tumor content in different samples (R 3.4.4 (R core Team (2018). R Foundation for Statistical Computing, Vienna, Austria). To lower the likelihood of type I error α was set at 0.001 and β at 0.004 (four-fold α) yielding a power of 0.996 [14–17].

### Ethics approval and consent to participate

This study was approved by the local ethics committee (Ethikkommission der Landesärztekammer Baden-Württemberg, F-2010-030) and undertaken in accordance with national law, institutional ethical standards, and the Helsinki Declaration. Written informed consent was provided either by the patient or a legally competent next of kin prior to the first study specific intervention.

## Results

### Patients and tumors

**Fig. 2:**
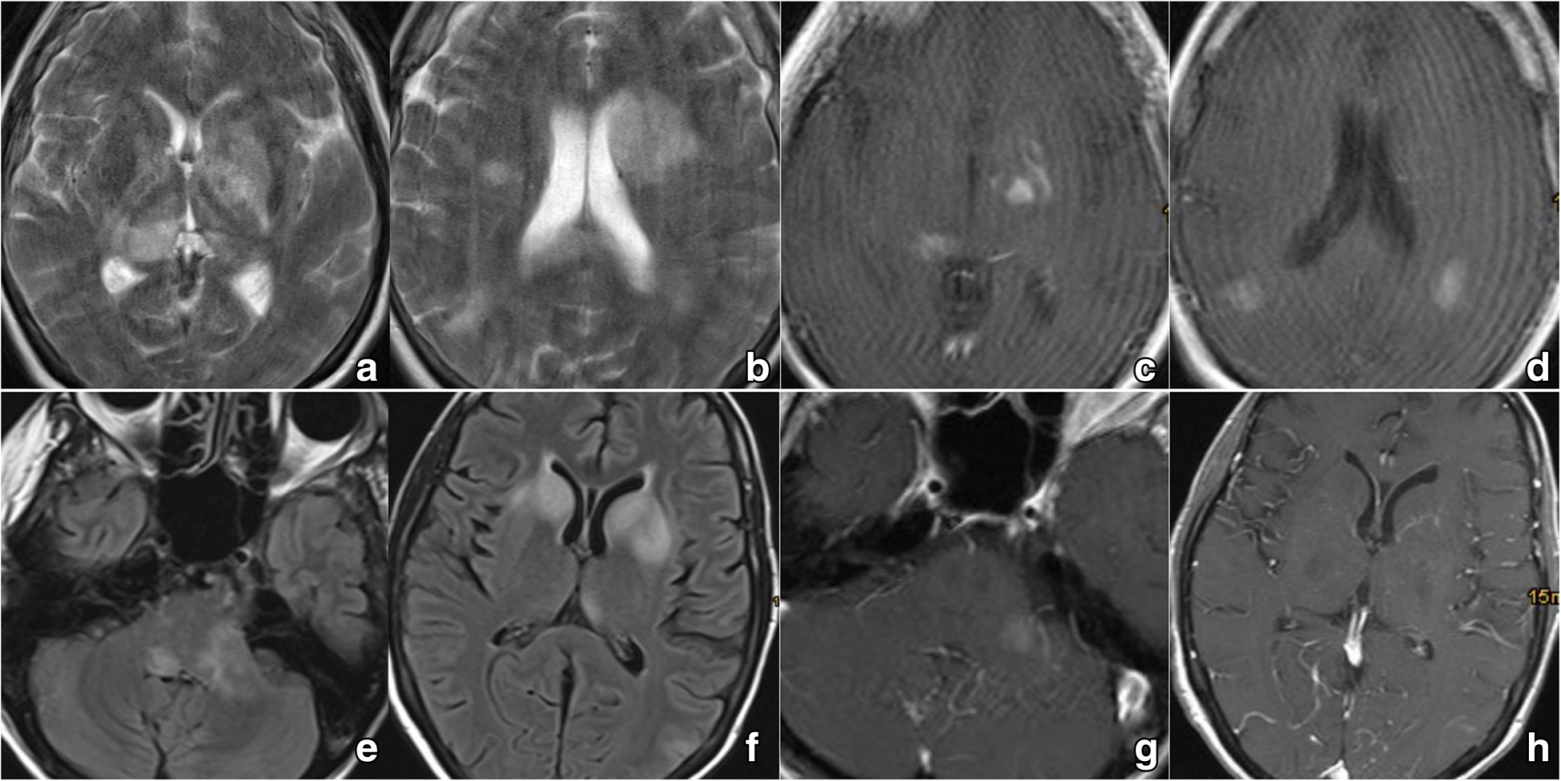
Cranial imaging of # 1, 2: **a, b**/**e, f**) MRI T2 flair showing multifocal hyperintense lesions with contact to the CSF space in both patients; **c, d**/**g, h**) MRI T1 with contrast showing only sparse enhancement in both patients

Six patients were recruited to test the feasibility of the applied techniques in patients with a central nervous system malignancy (mean age: 66.8 years, all female). Patient characteristics are displayed in Table 1. Representative images of the respective tumors are shown in Figs. 2 (patients #1 & #2) and 3 (patients #3–6), illustrating their location within the CNS and contact to the CSF space. CNSL was chosen because of routine necessity for lumbar puncture, for ethical reasons no study specific lumbar punctures were performed.

**Table 1:** Patient and Tumor characteristics

Nr.	Age Range	Symptoms	Duration of symptoms	Tumor location	in contact with CSF space	Contrast enhancement	Relevant patient history
1	70–79	general weakness, decline in overall health	few weeks	multifocal (left basal ganglia, right thalamus, hemispheres)	yes	Sparce, partial	no immunosuppression
2	40–49	general weakness, decline in overall health	few weeks	multifocal (basal ganglia, brainstem / medulla, cerebellar peduncles)	yes	Sparce, partial	HIV positive, previous EBV infection
3	60–69	gait disturbance, halluzinations	1,5 weeks	right basal ganglia	yes	Strong, homogenous	Melanoma of the scalp 4 years prior (excision only, no radiation/ chemo)
4	70–79	syncope, left hemiparesis, reduced alertness and arousal	2 weeks	bilateral basal ganglia, velum interpositum	yes	Strong, homogenous	no immunosuppression
5	70–79	right hemiparesis, reduced alertness, general weakness	3 weeks	left basal ganglia	yes	Strong, homogenous	no immunosuppression
6	70–79	right hand weakness, speech arrest	4 weeks	No tumor (contrast-enhancing meninges, left periventricular changes in T2 flair images)	yes	Strong (meninges)	systemtic B-cell lymphoma, full remission, no recurrence after 6 cycles of R-CHOP 8 years prior; rheumatoid arthritis (MTX-therapy)

**Fig. 3:**
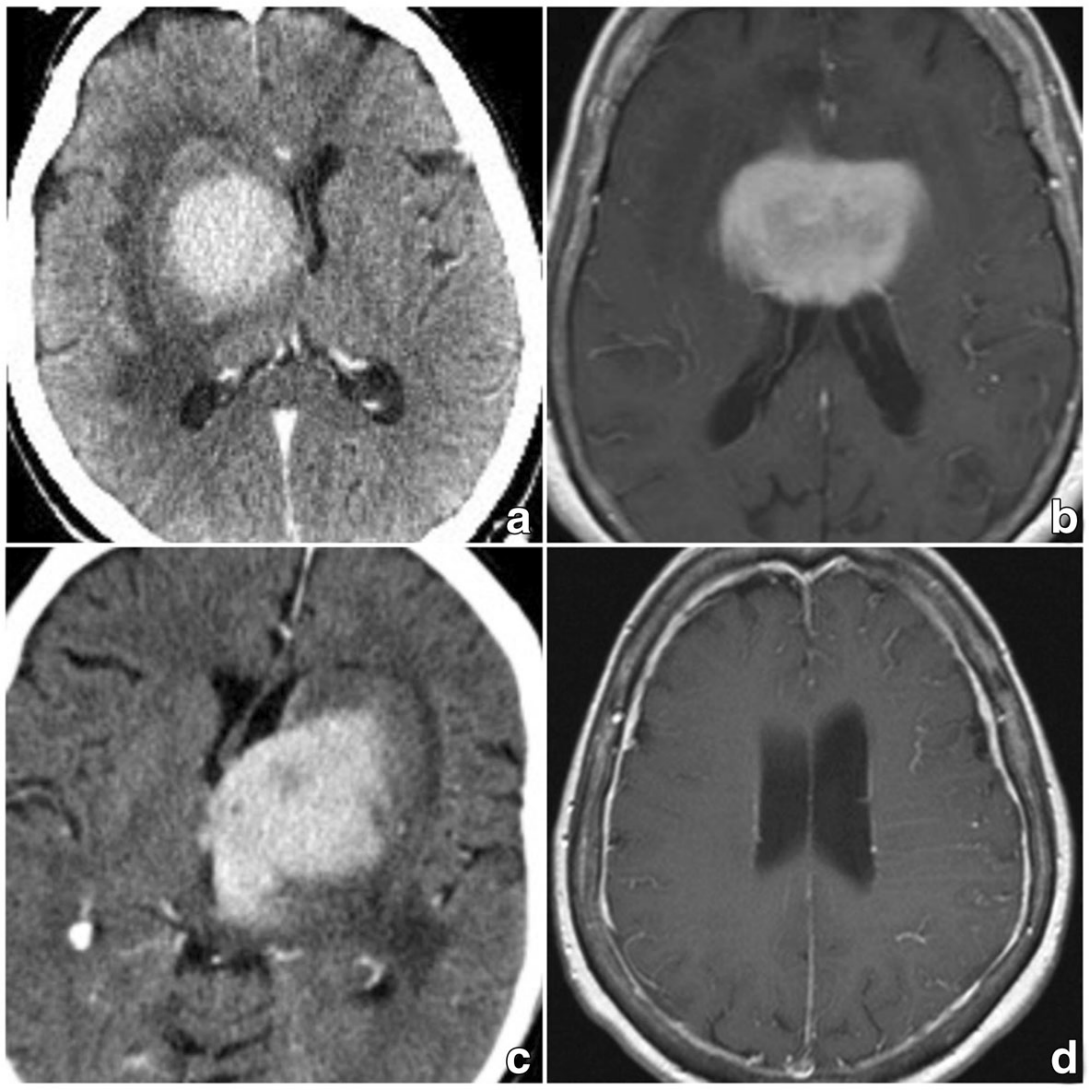
Cranial imaging of #3–6: **a**) CT with contrast (#3), **b**) MRI T1 with contrast, **c**) CT with contrast (#5); In contrast to the images in Fig. 2, homogenous, strong contrast enhancement is visible. **d**) MRI T1 with contrast (#6) showing homogenously thickened meninges and hypervascularity over the left hemisphere

### Pathology

CNSL was confirmed in 5 patients (83.3%) with identical pathologic diagnosis and microscopic appearance. Patient #6, who had a history of peripheral lymphoma, was found to have non-malignant hypervascularity of unknown significance.

Routine microscopic evaluation of CSF revealed tumor cells in 2 samples (*n* = 2/5, 40% of confirmed CNSL).

### Next-generation sequencing analysis of tumor tissue and individual mutation pattern

Somatic mutations were found in all 5 CNSL-samples (sensitivity: 100%). No mutation was detected in tissue sample #6 (specificity: 100%).

**Fig. 4:**
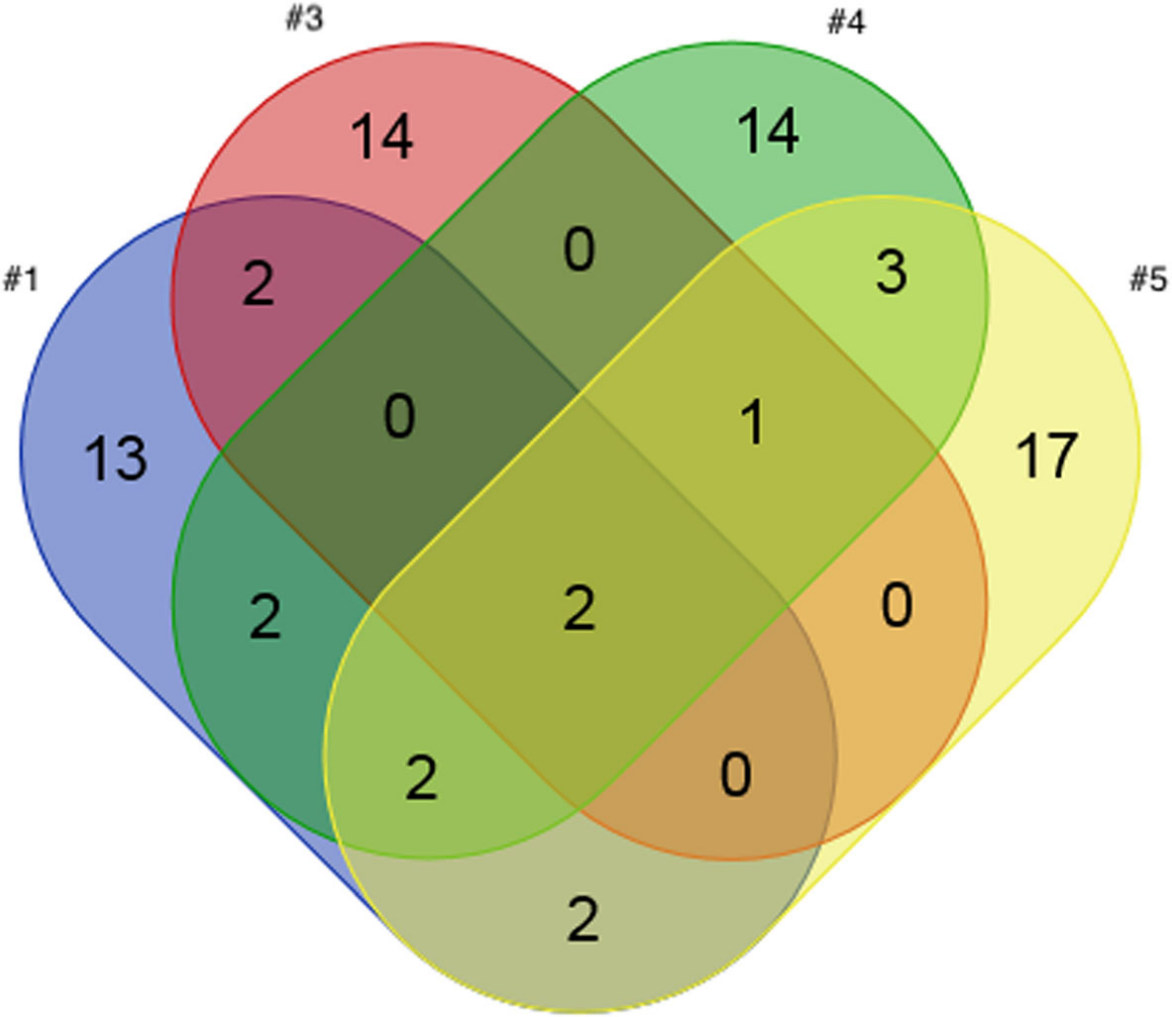
Venn diagram: Overlap of mutation profiles of tumor samples #1, 3, 4, 5. Note that this diagram shows the mutated genes. Therefore, the total number differs from Table 2, which shows all somatic mutations (2 independent mutations may occur within the same gene). Large copy number aberrations (CNAs) were not analyzed genome-wide, as the design of the NGS panel is optimized for the detection of single nucleotide variants (SNVs) and small copy number aberrations. All genes are listed in Table 3, their details are listed in the supplement (Additional file 1). Patients 2 and 6 were excluded from this diagram: The lymphoma sample #2 shows a divergent somatic mutation pattern (only 1 somatic mutation in MDM2) than the 4 other lymphomas and patient 6 had no somatic mutations at all, which is in accordance with the histopathologic diagnosis of absent malignancy. The mean coverage was >1000x in all tumor samples (ultra-deep sequencing)

Tumors #1, 3–5 demonstrated a high mutational load yielding mutations in some of the most commonly affected genes in lymphomas (TP53, ATM, KMT2D, STAT3, PAX5, CREBBP, CARD11) [18] (Tables 2, 3, Fig. 4; Additional file 1: section 4 – complete list of mutations).

**Table 2:** Results of Routine Pathologic Evaluation, Tumor Tissue Analysis und Analysis of cfDNA

Nr.	Routine Pathologic Evaluation		Tumor Tissue – NGS Panel Analysis			cfDNA - Plasma		cfDNA – CSF	
	*Histopathologic Diagnosis*	*Tumor cells in CSF*	*Number of somatic mutations* ^a^	*Selected Mutation for Assay (missense)*	*MAF (TC)*	*Tumor cfDNA*	*MAF*	*Tumor cfDNA*	*MAF*
1	Primary B-Cell Lymphoma	no	24	*EP400*-c.7792G > A (Chr.12)	0.32^b^ (50%)	no^d^	n.a.^d^	no^d^	n.a. ^d^
2	Primary B-Cell Lymphoma	no	1	*MDM2*-c.4G > A (Chr.12)	0.34^b^ (50%)	no^d^	n.a. ^d^	no^d^	n.a. ^d^
3	Primary B-Cell Lymphoma	no	20	*TP53*-c.845G > A (Chr.17)	0.44 (90%)	yes	0.04	yes	0.49
4	Primary B-Cell Lymphoma	yes	26	*BCL10*-c.677G > C (Chr.1)	0.47 (90%)	yes	0.004	yes	0.47
5	Primary B-Cell Lymphoma	yes	32	*ETV6*-c.1196G > A (Chr.12)	0.52^b,c^ (50%)	yes	0.02	yes	0.99 ^b,c^
6	Unspecific Hypervascularity/No malignancy	no	0	n.a.	n.a.	n.a.	n.a.	n.a.	n.a.

^a^2 independent somatic mutations within the same gene are counted as 2 mutations. Complex somatic aberrations on the same allele are counted as 1 mutation

^b^Loss of wt allele

^c^Additional duplication of the mutated allele

^d^Isolation of cfDNA failed in these samples, probably due to the isolation kit (see text)

Nomenclature of the mutations is according to NM_015409.4 (*EP400*), NM_002392.5 (*MDM2*), NM_001126114.2 (*TP53*), NM_003921.4 (*BCL10*), NM_001987.4 (*ETV6*)

*TC* tumor content (calculated based on the NAFs of various somatic mutations and single nucleotide variants both present in tumor and reference tissue)

*MAF* mutant allele frequency = the frequency with which the mutated allele occurs in the sequencing (1 equals 100%). The observed frequencies are influenced by the tumor content and do not correlate directly with the mutation frequency in the tumor (real frequency = tumor content x MAF). The MAF is also influenced by copy number aberrations

**Table 3:** mutated genes in tumor samples #1, 3, 4, 5

sample number	number of (shared) affected genes	by mutation affected genes
1,3,4,5	2	MYD88^a^, PIM1
1,4,5	2	BCL2^a^, ETV6
3,4,5	1	KMT2D
1,3	2	LPHN3, PRDM1
1,4	2	CD79B^a^, SOCS1
1,5	2	IRF4, MYC^a^
4,5	3	FOXB1, LRP1B^c^, HLA-B
1	13	LTF, EPHA5, EP400, SYNE1, BLNK, STAT3^a^, FES, SEPT9, POLR3A, DPYD, TFE3^b^, CSMD3, FAT1
3	14	KIT^a^, MAGI1, TP53^a^, ASXL1^a^, SETD2^a^, IDH2^a^, MTRR, PBRM1, BCR, MN1, RNF213, TOP1^a^, ATM^a^, FANCM^a^
4	14	CDKN1B^a^, PAX5, FOXO1, MCL1^a^, PTPRT, CARD11^c^, PPM1D, DST, BCL10, TCL1A, FN1, HSP90AA1^a^, NIN, SLCO1B1
5	17	HSP90AB1^a^, ARID5B^a^, ETS1, ERBB4^a^, CCND2^a^, HLA-C, ITGB2, EPHA3, BCL6, TBL1XR1, PCBP1, RECQL4, CREBBP, STAT4^a^, MLLT3, KEAP1, BTK^c^

^a^specific mutations/CNVs/increased expression of these genes qualify for targetable therapies (incl. Preclinical compounds)

^b^Mutations in these genes may alter pharmacokinetics of drugs (Pharmacogenomics)

^c^Mutations in these genes are prohibitive for certain targetable therapies [53]

*PIM1* and *MYD88* were altered in each lymphoma. MYD88 is the most frequently somatically mutated gene in brain lymphomas [18]. PIM1 is expressed primarily in B-lymphoid and myeloid cell lines and is overexpressed in hematopoietic malignancies [18, 19]. The analysis of tumor #2 revealed just one mutation (MDM2), seen in this one only. In contrast to the other samples, the harboring patient was HIV+ with serologic evidence of an EBV-infection, a known cause of Burkitt-lymphomas, which frequently harbor mutations in the ARF/MDM2-gene [20]. Thus, the divergent homogenous molecular profile may be attributable to the infections and their potential role in tumor genesis, despite the identical microscopic appearance of all tumors. The Venn diagram (Fig. 4), illustrating the variety of and yet overlapping mutation patterns among the different tumors (Table 3) was drawn using an online tool (http://bioinformatics.psb.ugent.be/webtools/Venn/).

### cfDNA analysis

**Fig. 5:**
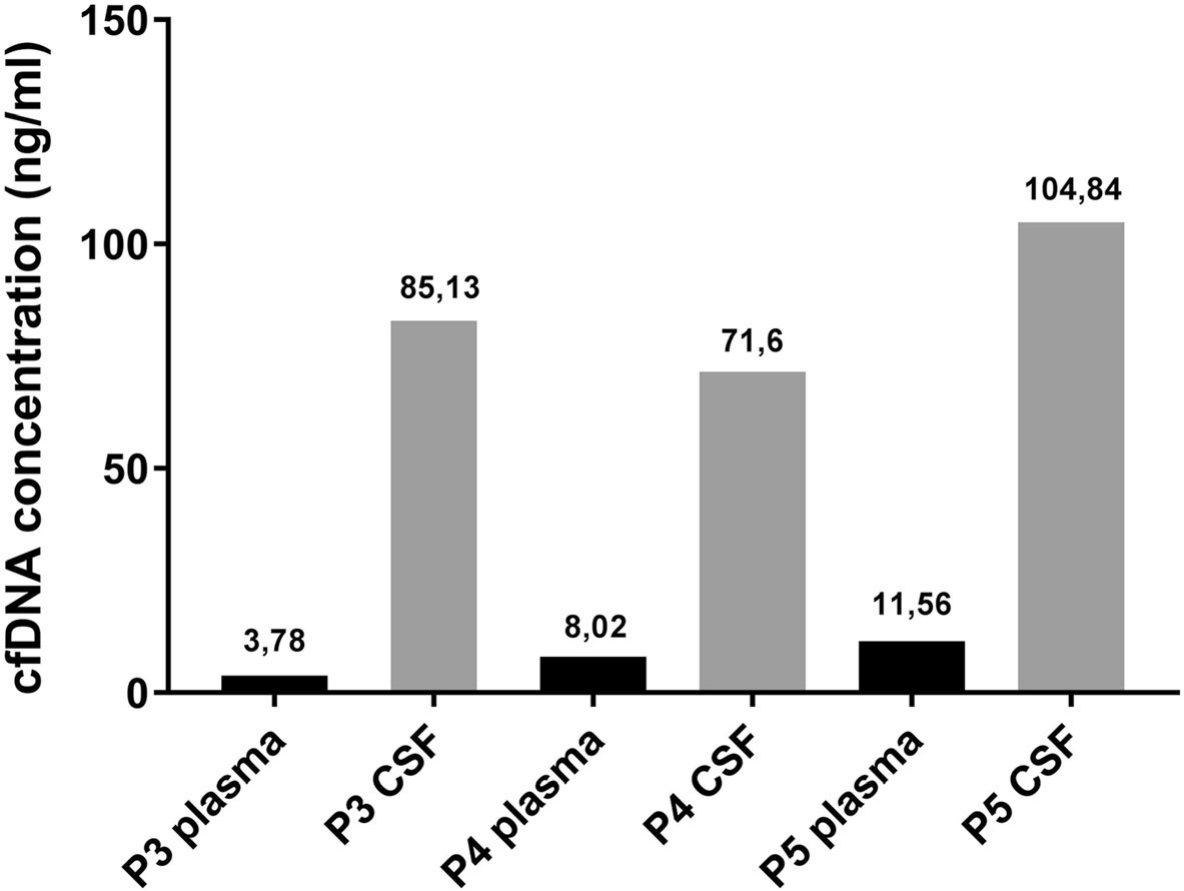
cfDNA concentration in plasma vs. CSF. Fluorometric measurement of cfDNA concentration in CSF and plasma. For plasma and csf samples #1 and #2 no cfDNA could be detected, most probably due to technical reasons (explanation see text)

cfDNA concentration was higher in all CSF- than in the corresponding plasma-samples (fluorometric quantification) (Table 2, Fig. 5). Contamination of CSF with cellular genomic DNA was excluded by fragment size distribution analysis yielding no high molecular weight DNA (Additional file 1: section 5, Additional file 4: Figure S3).

**Fig. 6:**
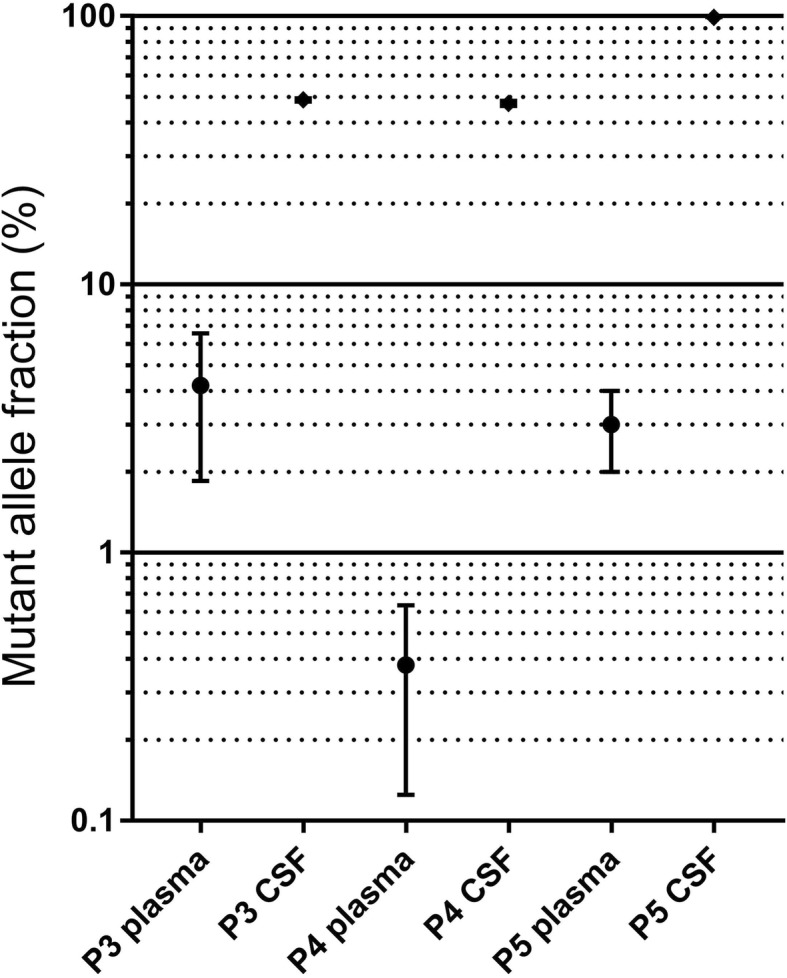
Mutant Allele Fraction in plasma and CSF of patients with feasible extraction. For plasma and csf samples #1 and #2 no cfDNA could be detected, most probably due to technical reasons (explanation see text)

Extraction was not feasible in the first two samples prior to the change in cfDNA extraction kits. ctDNA was detected in all samples from patients with confirmed tumor and feasible cfDNA-extraction (*n* = 3). CSF-derived cfDNA was almost exclusively of tumoral origin with only small portions of tumor-derived cfDNA in the plasma as indicated by the MAF (Table 2, Fig. 6).

The two cases (40%), in which cfDNA could neither be detected in blood nor in CSF, were pretreated with a different kit for DNA extraction as described above, which had internally proven to not be optimal for this purpose in other unrelated cases. Because no cfDNA could be detected, a failure of the kit is assumed, despite a report by Wang et al. describing the possible influence of a tumor’s location within the CNS on ctDNA detection in CSF (e.g. tumors completely surrounded by parenchyma) [21]. In these particular two cases, tumors did show a different contrast enhancement (less homogenous) indicating a different breakdown of the blood-brain barrier/tumor perfusion, yet all tumors were in contact with the CSF space.

### Required input material and time for analysis

For the present investigation 20 ml whole blood (10 ml EDTA tube, 10 ml Cell-Free DNA BCT® Tubes (Streck, Omaha, NE, USA)) and 1-4 ml CSF were used, yielding 3-4 ml of blood and 0.9–2.7 ml of CSF after pretreatment. The exact amount of extracted cfDNA is displayed in Fig. 5. The amount of CSF varied, because no study specific-CSF drawings were performed, only material left from routine diagnostics was used.

When planning the study and beginning the analysis we were not aware of the amount of required cfDNA necessary for detection of relevant mutations and their respective concentration, thus all material was used for best possible results, especially after the first two negative results. An NGS panel analysis from cfDNA in this cohort was therefore not feasible. From other internal analyses in unrelated cases, we know that about 200 ng CSF would have been necessary for a complete panel analysis. Today 10-20 ng of cfDNA is sufficient allowing enrichment of commonly mutated genes and detection of mutations. Considering the rapid advances in NGS analysis over the past few years and applying current standards, panel diagnostics should have been feasible in all samples, except for plasma of patient 3. At the time of our laboratory investigation, only CSF of patient #3 yielded a high enough concentration of cfDNA for a panel analysis.

Enrichment took about 1 h, droplet generation 30 min, thermocycling 1.33 h and droplet analysis 1 h. Sequencing required 17 h. For analysis of data, usually 4 h were necessary. Thus, approximately 24-28 h are necessary for complete analysis. Because for NGS panel analysis several samples are collected for efficient use of resources, overall analysis currently takes 3 weeks, aiming at 2 weeks.

## Discussion

Personalized approaches are advancing in oncology and therapies are adapted to the genetic profile of each tumor. Targeted therapies, aiming at specific mutations, have greatly improved treatment and survival in some cancers [22, 23]. Kinugasa et al. were able to perform K-ras genotyping in pancreatic cancer from plasma cfDNA, which had a significant impact on the prognosis [24]. Diaz et al. and Gray et al. detected mutations causing targeted-therapy resistance even prior to apparent disease progression [25, 26]. Furthermore, the genetic variance has become important for estimating prognosis and evaluating patients’ risk-benefit-profile for standard treatment (e.g. gliomas [27]).

New NGS-technologies allow the detection of a great variety of somatic mutations instead of just screening for known “hotspot”-mutations [28], improving survival without adding to the financial burden in cancer care [29]. But the acquisition of input material is often associated with biopsies and their inherent risk of surgical complications, which can be especially devastating in cerebral malignancies. Therefore new approaches to detect specific, targetable mutations and improve surveillance for central nervous system malignancies prior to visible progression as described for colorectal cancer [25] and melanoma [26] are desirable. Already several molecular markers and circulating proteins have been identified using peripheral blood samples in patients with primary brain tumors [10–12]. Even though with the standardized NGS-procedures the respective MAF should exceed 5% to allow robust variant detection. Thus the blood-brain barrier may hinder detection of the required amount of ctDNA in plasma as indicated by our results, which are in accordance with others [10, 13]. However, sequencing of CSF-derived cfDNA could yield therapeutically useful results, as the respective cfDNA is almost exclusively of tumoral origin. Thereby one of the limitations encountered in the analysis of tissue biopsies can be overcome, because with tissue biopsies, only DNA from small parts of a genetically heterogeneous tumor are evaluated while circulating DNA is derived from multiple parts of the tumor. Novel NGS library preparations lowered the amount of required DNA input material to ≤10 ng, which should enable NGS-analysis of cfDNA without needing large CSF-quantities. This study was intended to prove that tumor-specific mutations can be detected applying the protocol outlined above. In future studies, it is now possible to investigate whether NGS panel sequencing is feasible using this approach. A specific mutation had to be selected to allow interpretation of the results. Applying a broad approach using panel diagnostics from the beginning with the potential for negative results may have led to the assumption that cfDNA is not detectable at all. Alternatively, designing an assay targeting the most commonly affected genes should be feasible. In accordance with our findings, the majority of samples in the cosmic database positive for PIM1 were also MYD88 positive [18], therefore these two could serve as a basis for an assay. But as shown by Fontanilles et al., using a targeted panel, in only 32% of cases somatic mutations were detected in ctDNA, yielding a sensitivity of 24% for analysis of plasma of CNSL patients [30] underlining the superiority of ctDNA derived from CNS and broad panel analysis.

An interesting finding in this small cohort was patient #2, which highlights the importance genetic analysis may have in general when planning tumor treatment and discussing prognosis. All tumors were classified as B-cell lymphomas without any specification as to their origination. But the divergent homogenous molecular profile of patient #2’s tumor may be attributable to the infections (HIV and EBV) and their potential role in tumor genesis. Unfortunately, the patient died from a deep-seated bleeding after biopsy not amenable to surgery, thus whether the response to treatment would have been different cannot be reported.

Despite the possibilities in other entities [8], diagnosing CNSL based on their genetic profile from ctDNA alone applying this minimally invasive approach with less procedural risks compared to needle biopsies, is not possible, yet. To date, too little is known about CNSL’s genetic profile in general, even though several mutations, which have also been identified in this study, have been described before [18, 30–34]. One study has already demonstrated that CNSL could be differentiated from glioblastoma based on analysis of ctDNA [30].

Nonetheless, knowing a tumor’s mutation-pattern may alter its treatment by omitting evidently ineffective or adding specific therapies, such as targeted / immunologic approaches, as in other cancers [35]. In Table 3, mutations identified in this small cohort amenable to targeted therapies, those altering response to treatment and mutations prohibiting certain targeted therapies are highlighted. For example, certain MYC, TP53 and IDH mutations in various tumors are investigated as targets for specific treatments [36–50]. But despite increasing reports on possible therapies, currently, they should be discussed in interdisciplinary molecular tumor boards to consider previous treatments and the patient-specific mutation profile in the decision-making process, since different mutations in one gene may have different biological effects (e.g. activating vs. inhibiting). Additionally, targeted therapies do not yet comprise standard treatments in most tumors and are therefore often off-label.

Furthermore, repetitive ctDNA-evaluation aiming at tumor-specific somatic mutations could aid in treatment surveillance in central nervous system tumors.

### Limitations

With only a small number of cases evaluated in this feasibility investigation, the conclusions, as well as future outlooks, have to be interpreted with caution. Future research is needed to identify lymphoma-specific mutational patterns as has been possible for glioblastoma in recent years [51, 52] and to test feasibility for other entities such as metastasis and brain stem gliomas. Knowing about required input material now, future investigations should also focus on investigating the feasibility of panel analyses to detect a broader spectrum of possible treatment relevant mutations. To guide future studies on this topic, we determined a sample size of > 3 to yield statistically reliable results of comparisons of tumor content between plasma and CSF, as well as a sample size > 6 for comparisons of concentrations of cfDNA, based on the measured fractions of ctDNA in this investigation (α = 0.001, β = 0.004, power = 0.996). The superiority of CSF in detecting ctDNA could thus be assumed. Being aware of the difficulties next generation sequencing imposes on statistical analysis (e.g. multiple testing) [16, 17], we have focused on tumor content and ctDNA concentration in general in our sample size determination and not on detection of changes in specific genes due to the heterogeneity of affected genes in this small study sample.

## Conclusion

Analysis of cfDNA from CSF in central nervous system tumors is feasible. Despite the blood-brain barrier, small amounts of ctDNA could be detected in the plasma, but are insufficient for reliable diagnostics. This investigation provides evidence that molecular characterization of CNSL can be achieved by analysis of CSF-derived cfDNA, but further investigations are necessary. Knowing a tumor’s specific mutation pattern can aid in treatment planning and surveillance.

## Additional files

Additional file 1: Supplemental digital content: 1. Genes covered in the TUM01 NGS tumor panel: list of all genes covered by the panel. 2. Primer and Probes for TaqMan-Assays: sequence of primers used for TaqMan-Assays. 3. Digitial droplet PCR: detailed description of PCR as performed for this study, illustrated by Additional file 2: Figure S1 and Additional file 3: Figure S2. 4. Specific Mutation Pattern of Tumors: list of all detected mutations in the tumors of this study population with detailed explanation on position, kind of mutation, amino acid change and nucleotide change. 5. BioAnalyzer – cfDNA: description of fragment size distribution, illustrated by Additional file 4: Figure S3. (DOCX 60 kb)

Additional file 2: **Figure S1.** ddPCR Assays optimization (blood). DNA from the respective blood sample was used as input material to determine the optimal PCR annealing temperature for the detection of the wildtype allele (TP53 .845G) (Chr.17)). (TIFF 1210 kb)

Additional file 3: **Figure S2.** ddPCR Assays optimization (tumor). DNA from the respective tumor tissue was used as input material to determine the optimal PCR annealing temperature for the detection of the mutant allele (TP53 845A) (Chr.17). (TIFF 1239 kb)

Additional file 4: **Figure S3.** Fragment Size Distribution. Typical image of a fragment size distribution analysis of circulating DNA (cfDNA) after isolation from a blood sample. (TIFF 912 kb)

## Abbreviations

cfDNA: Circulating free DNA
CNS: Central nervous system
CNSL: Central nervous system lymphoma
CSF: Cerebrospinal fluid
ctDNA: Circulating tumor DNA
dPCR: Digital polymerase chain reaction
EDTA: Ethlyendiamintetraacetic acid
MAF: Mutant allele frequency
NGS: Next generation sequencing
TC: Tumor content

## References

[1] Ferreri AJ, Marturano E (2012). Primary CNS lymphoma. Best Pract Res Clin Haematol.

[2] Feiden W, Milutinovic S (2002). Primary CNS lymphomas. Morphology and diagnosis. Pathologe.

[3] Verploegh IS, Volovici V, Haitsma IK, Schouten JW, Dirven CM, Kros JM, Dammers R (2015). Contemporary frameless intracranial biopsy techniques: might variation in safety and efficacy be expected?. Acta Neurochir.

[4] Benesova L, Belsanova B, Suchanek S, Kopeckova M, Minarikova P, Lipska L, Levy M, Visokai V, Zavoral M, Minarik M (2013). Mutation-based detection and monitoring of cell-free tumor DNA in peripheral blood of cancer patients. Anal Biochem.

[5] Diaz LA Jr, Bardelli A. Liquid biopsies: genotyping circulating tumor DNA. J Clin Oncol. 2014;32(6):579-86. 10.1200/JCO.2012.45.2011. Epub 2014 Jan 21. 10.1200/JCO.2012.45.2011 PMC4820760 24449238

[6] Marzese DM, Hirose H, Hoon DS (2013). Diagnostic and prognostic value of circulating tumor-related DNA in cancer patients. Expert Rev Mol Diagn.

[7] Koyanagi K, Mori T, O'Day SJ, Martinez SR, Wang HJ, Hoon DS (2006). Association of circulating tumor cells with serum tumor-related methylated DNA in peripheral blood of melanoma patients. Cancer Res.

[8] Diehl F, Li M, Dressman D, He Y, Shen D, Szabo S, Diaz LA, Goodman SN, David KA, Juhl H (2005). Detection and quantification of mutations in the plasma of patients with colorectal tumors. Proc Natl Acad Sci U S A.

[9] Daniotti M, Vallacchi V, Rivoltini L, Patuzzo R, Santinami M, Arienti F, Cutolo G, Pierotti MA, Parmiani G, Rodolfo M (2007). Detection of mutated BRAFV600E variant in circulating DNA of stage III-IV melanoma patients. Int J Cancer.

[10] Best MG, Sol N, Zijl S, Reijneveld JC, Wesseling P, Wurdinger T (2015). Liquid biopsies in patients with diffuse glioma. Acta Neuropathol.

[11] Weaver KD, Grossman SA, Herman JG (2006). Methylated tumor-specific DNA as a plasma biomarker in patients with glioma. Cancer Investig.

[12] Liu BL, Cheng JX, Zhang W, Zhang X, Wang R, Lin H, Huo JL, Cheng H (2010). Quantitative detection of multiple gene promoter hypermethylation in tumor tissue, serum, and cerebrospinal fluid predicts prognosis of malignant gliomas. Neuro-Oncology.

[13] Shi W, Lv C, Qi J, Zhao W, Wu X, Jing R, Wu X, Ju S, Chen J (2012). Prognostic value of free DNA quantification in serum and cerebrospinal fluid in glioma patients. J Mol Neurosci.

[14] Banerjee A, Chitnis UB, Jadhav SL, Bhawalkar JS, Chaudhury S (2009). Hypothesis testing, type I and type II errors. Ind Psychiatry J.

[15] Akobeng AK (2016). Understanding type I and type II errors, statistical power and sample size. Acta Paediatr.

[16] Mudge JF, Martyniuk CJ, Houlahan JE (2017). Optimal alpha reduces error rates in gene expression studies: a meta-analysis approach. BMC Bioinformatics.

[17] Bi R, Liu P (2016). Sample size calculation while controlling false discovery rate for differential expression analysis with RNA-sequencing experiments. BMC Bioinformatics.

[18] Cosmic - Catalogue of somatic mutations in cancer [http://cancer.sanger.ac.uk/cosmic/browse/tissue-in=t&sn=haematopoietic_and_lymphoid_tissue&ss=all&hn=lymphoid_neoplasm. Accessed 14 Apr 2018.

[19] NCBI Gene database [https://www.ncbi.nlm.nih.gov/gene/5292]. Accessed 14 Apr 2018.

[20] Lindstrom MS, Wiman KG (2002). Role of genetic and epigenetic changes in Burkitt lymphoma. Semin Cancer Biol.

[21] Wang Y, Springer S, Zhang M, McMahon KW, Kinde I, Dobbyn L, Ptak J, Brem H, Chaichana K, Gallia GL (2015). Detection of tumor-derived DNA in cerebrospinal fluid of patients with primary tumors of the brain and spinal cord. Proc Natl Acad Sci U S A.

[22] Pinilla-Ibarz J, Sweet KL, Corrales-Yepez GM, Komrokji RS (2016). Role of tyrosine-kinase inhibitors in myeloproliferative neoplasms: comparative lessons learned. Onco Targets Ther.

[23] Zhang B, Hurvitz S (2016). Long-term outcomes of neoadjuvant treatment of HER2-positive breast cancer. Clin Adv Hematol Oncol.

[24] Kinugasa H, Nouso K, Miyahara K, Morimoto Y, Dohi C, Tsutsumi K, Kato H, Matsubara T, Okada H, Yamamoto K (2015). Detection of K-ras gene mutation by liquid biopsy in patients with pancreatic cancer. Cancer.

[25] Diaz LA, Williams RT, Wu J, Kinde I, Hecht JR, Berlin J, Allen B, Bozic I, Reiter JG, Nowak MA (2012). The molecular evolution of acquired resistance to targeted EGFR blockade in colorectal cancers. Nature.

[26] Gray ES, Rizos H, Reid AL, Boyd SC, Pereira MR, Lo J, Tembe V, Freeman J, Lee JH, Scolyer RA (2015). Circulating tumor DNA to monitor treatment response and detect acquired resistance in patients with metastatic melanoma. Oncotarget.

[27] Weller M, Reifenberger G, Tonn JC, Wick W. Gliome: Aktuelle Entwicklungen in der Diagnostik und Therapie. Dtsch Arztebl. 2016;113(6):18. 10.3238/PersOnko/2016.02.12.04.

[28] Frampton GM, Fichtenholtz A, Otto GA, Wang K, Downing SR, He J, Schnall-Levin M, White J, Sanford EM, An P, et al. Development and validation of a clinical cancer genomic profiling test based on massively parallel DNA sequencing. Nat Biotechnol. 2013;31(11):1023–31. 10.1038/nbt.2696. Epub 2013 Oct 20. 10.1038/nbt.2696 PMC5710001 24142049

[29] Haslem DS, Van Norman SB, Fulde G, Knighton AJ, Belnap T, Butler AM, Rhagunath S, Newman D, Gilbert H, Tudor BP, et al. A retrospective analysis of precision medicine outcomes in patients with advanced Cancer reveals improved progression-free survival without increased health care costs. J Oncol Pract. 2017;13(2):e108-19. 10.1200/JOP.2016.011486. Epub 2016 Oct 31. 10.1200/JOP.2016.011486 PMC5455156 27601506

[30] Fontanilles M, Marguet F, Bohers E, Viailly PJ, Dubois S, Bertrand P, Camus V, Mareschal S, Ruminy P, Maingonnat C, et al. Non-invasive detection of somatic mutations using next-generation sequencing in primary central nervous system lymphoma. Oncotarget. 2017;8(29):48157–68. 10.18632/oncotarget.18325. 10.18632/oncotarget.18325 PMC5564634 28636991

[31] Bruno A, Boisselier B, Labreche K, Marie Y, Polivka M, Jouvet A, Adam C, Figarella-Branger D, Miquel C, Eimer S (2014). Mutational analysis of primary central nervous system lymphoma. Oncotarget.

[32] Todorovic Balint M, Jelicic J, Mihaljevic B, Kostic J, Stanic B, Balint B, Pejanovic N, Lucic B, Tosic N, Marjanovic I, et al. Gene mutation profiles in primary diffuse large B cell lymphoma of central nervous system: next generation sequencing analyses. Int J Mol Sci. 2016;17(5). 10.3390/ijms17050683 PMC4881509 27164089

[33] Deckert M, Montesinos-Rongen M, Brunn A, Siebert R. Systems biology of primary CNS lymphoma: from genetic aberrations to modeling in mice. Acta Neuropathol. 2014;127(2):175–88. 10.1007/s00401-013-1202-x. Epub 2013 Nov 16. 10.1007/s00401-013-1202-x 24240734

[34] Lim DH, Kim WS, Kim SJ, Yoo HY, Ko YH (2015). Microarray gene-expression profiling analysis comparing PCNSL and non-CNS diffuse large B-cell lymphoma. Anticancer Res.

[35] Kelly CM, Janjigian YY (2016). The genomics and therapeutics of HER2-positive gastric cancer-from trastuzumab and beyond. J Gastrointest Oncol.

[36] Mollaoglu G, Guthrie MR, Bohm S, Bragelmann J, Can I, Ballieu PM, Marx A, George J, Heinen C, Chalishazar MD (2017). MYC drives progression of small cell lung Cancer to a variant neuroendocrine subtype with vulnerability to Aurora kinase inhibition. Cancer Cell.

[37] Hook KE, Garza SJ, Lira ME, Ching KA, Lee NV, Cao J, Yuan J, Ye J, Ozeck M, Shi ST (2012). An integrated genomic approach to identify predictive biomarkers of response to the aurora kinase inhibitor PF-03814735. Mol Cancer Ther.

[38] Posternak V, Cole MD. Strategically targeting MYC in cancer. F1000Res. 2016;5. 10.12688/f1000research.7879.1. 10.12688/f1000research.7879.1 PMC4813636 27081479

[39] Sabnis HS, Somasagara RR, Bunting KD. Targeting MYC dependence by metabolic inhibitors in Cancer. Genes (Basel). 2017;8(4). 10.3390/genes8040114 PMC5406861 28362357

[40] Schuler PJ, Harasymczuk M, Visus C, Deleo A, Trivedi S, Lei Y, Argiris A, Gooding W, Butterfield LH, Whiteside TL (2014). Phase I dendritic cell p53 peptide vaccine for head and neck cancer. Clin Cancer Res.

[41] Saito H, Ando S, Morishita N, Lee KM, Dator D, Dy D, Shigemura K, Adhim Z, Nibu K, Fujisawa M (2014). A combined lymphokine-activated killer (LAK) cell immunotherapy and adenovirus-p53 gene therapy for head and neck squamous cell carcinoma. Anticancer Res.

[42] Synnott NC, Murray A, McGowan PM, Kiely M, Kiely PA, O'Donovan N, O'Connor DP, Gallagher WM, Crown J, Duffy MJ (2017). Mutant p53: a novel target for the treatment of patients with triple-negative breast cancer?. Int J Cancer.

[43] Bridges KA, Hirai H, Buser CA, Brooks C, Liu H, Buchholz TA, Molkentine JM, Mason KA, Meyn RE (2011). MK-1775, a novel Wee1 kinase inhibitor, radiosensitizes p53-defective human tumor cells. Clin Cancer Res.

[44] Vilgelm AE, Pawlikowski JS, Liu Y, Hawkins OE, Davis TA, Smith J, Weller KP, Horton LW, McClain CM, Ayers GD (2015). Mdm2 and aurora kinase a inhibitors synergize to block melanoma growth by driving apoptosis and immune clearance of tumor cells. Cancer Res.

[45] Li Z, Sun Y, Chen X, Squires J, Nowroozizadeh B, Liang C, Huang J (2015). p53 mutation directs AURKA overexpression via miR-25 and FBXW7 in prostatic small cell neuroendocrine carcinoma. Mol Cancer Res.

[46] Zawacka-Pankau J, Selivanova G (2015). Pharmacological reactivation of p53 as a strategy to treat cancer. J Intern Med.

[47] Zhao D, Tahaney WM, Mazumdar A, Savage MI, Brown PH (2017). Molecularly targeted therapies for p53-mutant cancers. Cell Mol Life Sci.

[48] Rohle D, Popovici-Muller J, Palaskas N, Turcan S, Grommes C, Campos C, Tsoi J, Clark O, Oldrini B, Komisopoulou E (2013). An inhibitor of mutant IDH1 delays growth and promotes differentiation of glioma cells. Science.

[49] Seystahl K, Gramatzki D, Roth P, Weller M (2016). Pharmacotherapies for the treatment of glioblastoma - current evidence and perspectives. Expert Opin Pharmacother.

[50] Sulkowski PL, Corso CD, Robinson ND, Scanlon SE, Purshouse KR, Bai H, Liu Y, Sundaram RK, Hegan DC, Fons NR, et al. 2-Hydroxyglutarate produced by neomorphic IDH mutations suppresses homologous recombination and induces PARP inhibitor sensitivity. Sci Transl Med. 2017;9(375). 10.1126/scitranslmed.aal2463. 10.1126/scitranslmed.aal2463 PMC5435119 28148839

[51] Ceccarelli M, Barthel FP, Malta TM, Sabedot TS, Salama SR, Murray BA, Morozova O, Newton Y, Radenbaugh A, Pagnotta SM (2016). Molecular profiling reveals biologically discrete subsets and pathways of progression in diffuse glioma. Cell.

[52] Brennan CW, Verhaak RG, McKenna A, Campos B, Noushmehr H, Salama SR, Zheng S, Chakravarty D, Sanborn JZ, Berman SH (2013). The somatic genomic landscape of glioblastoma. Cell.

[53] DGIdb - The Drug Gene Interaction Database [http://www.dgidb.org/search_interactions]. Accessed 14 Apr 2018.

