# Supplementary material for: Molecular tumor analysis and liquid biopsy: a feasibility investigation analyzing circulating tumor DNA in patients with central nervous system lymphomas
Source: BMC Cancer. 2019 Mar 1;19:192. doi: 10.1186/s12885-019-5394-x (PMC6397454; doi:10.1186/s12885-019-5394-x)
Supplement: Supplementary file 1 — Supplemental digital content: 1. Genes covered in the TUM01 NGS tumor panel: list of all genes covered by the panel. 2. Primer and Probes for TaqMan-Assays: sequence of primers used for TaqMan-Assays. 3. Digitial droplet PCR: detailed description of PCR as performed for this study, illustrated by Additional file 2: Figure S1 and Additional file 3: Figure S2. 4. Specific Mutation Pattern of Tumors: list of all detected mutations in the tumors of this study population with detailed explanation on position, kind of mutation, amino acid change and nucleotide change. 5. BioAnalyzer – cfDNA: description of fragment size distribution, illustrated by Additional file 4: Figure S3. (DOCX 60 kb) [file 12885_2019_5394_MOESM1_ESM.docx]

**Supplementary material**

**1. Genes covered in the TUM01 NGS tumor panel:**

*ABCB1, ABCC2, ABCG2, ABL1, ABL2, ACD, ACVR1, ACVR1B, AJUBA, AKT1,*

*AKT2, AKT3, ALK, AMER1, APC, AR, ARAF, ARFRP1, ARHGAP35, ARID1A,*

*ARID1B, ARID2, ARID5B, ARNT, ASXL1, ATF1, ATM, ATP1A1, ATP5B, ATR,*

*ATRX, AURKA, AURKB, AURKC, AXIN1, AXIN2, AXL, AZGP1, B2M, BAP1,*

*BARD1, BCL10, BCL11A, BCL11B, BCL2, BCL2L1, BCL2L2, BCL3, BCL6,*

*BCL9, BCOR, BCORL1, BCR, BIRC2, BIRC3, BIRC5, BLM, BMPR1A, BRAF,*

*BRCA1, BRCA2, BRD4, BRE, BRIP1, BTK, BTNL2, BUB1B, C11ORF30,*

*CARD11, CASP8, CBFB, CBL, CCDC6, CCND1, CCND2, CCND3, CCNE1,*

*CD1D, CD274, CD70, CD79A, CD79B, CD82, CDC27, CDC73, CDH1, CDH2,*

*CDK12, CDK4, CDK6, CDK8, CDKN1A, CDKN1B, CDKN2A, CDKN2B,*

*CDKN2C, CDX2, CEBPA, CEP57, CHD2, CHD4, CHEK1, CHEK2, CIC,*

*CKS1B, COL1A1, CREB1, CREBBP, CRKL, CRTC1, CSF1R, CTCF, CTNNA1,*

*CTNNB1, CUL3, CUL4B, CUX1, CYLD, CYP1A1, CYP1A2, CYP2A6, CYP2B6,*

*CYP2C19, CYP2C8, CYP2C9, CYP2D6, CYP2E1, CYP3A4, CYP3A5, DAXX,*

*DCC, DDB2, DDIT3, DDR2, DDX3X, DEK, DIAPH1, DICER1, DIS3, DNMT1,*

*DNMT3A, DOT1L, DPYD, DST, EGFR, EGR3, ELAC2, ELF3, EML4, ENG,*

*EP300, EPCAM, EPHA2, EPHA3, EPHA5, EPHA7, EPHB1, EPHB4, EPHB6,*

*EPHX1, ERBB2, ERBB3, ERBB4, ERCC1, ERCC2, ERCC3, ERCC4, ERCC5,*

*ERG, ERRFI1, ESR1, ETS1, ETV1, ETV4, ETV5, ETV6, EWSR1, EXT1,*

*EXT2, EZH1, EZH2, FAM175A, FAM46C, FANCA, FANCB, FANCC, FANCD2,*

*FANCE, FANCF, FANCG, FANCI, FANCL, FANCM, FAS, FAT1, FBXW7, FES,*

*FGF10, FGF14, FGF19, FGF23, FGF3, FGF4, FGF6, FGFBP1, FGFR1,*

*FGFR2, FGFR3, FGFR4, FH, FLCN, FLI1, FLT1, FLT4, FN1, FOXA1, FOXA2,*

*FOXE1, FOXL2, FOXO1, FOXO3, FOXP1, FOXQ1, FRS2, FUBP1, FUS,*

*G6PD, GABRA6, GALNT12, GATA1, GATA2, GATA3, GATA4, GATA6, GDNF,*

*GID4, GLI1, GNA11, GNA13, GNAQ, GNAS, GOT1, GPC3, GPR124, GRIN2A,*

*GRM3, GSK3B, GSTM1, GSTP1, GSTT1, GUSB, H3F3A, H3F3B, HGF, HIF1A,*

*HIST1H3B, HLA-A, HLA-B, HLA-C, HLF, HMGA2, HNF1A, HNF1B, HOXA9,*

*HOXB13, HRAS, HSD3B1, HSP90AA1, HSP90AB1, IDH1, IDH2, IGF1R, IGF2,*

*IGF2R, IKBKB, IKBKE, IKZF1, IL2, IL21R, IL6ST, IL7R, ING1, ING4, INHBA,*

*INPP4B, INPPL1, IRF2, IRF4, IRF6, IRS2, ITGB2, ITK, JAK1, JAK2, JAK3,*

*JUN, KAT6A, KCNJ5, KDM5A, KDM5C, KDM6A, KDR, KEAP1, KEL, KIAA1549,*

*KIT, KLF4, KLF6, KLHL6, KMT2A, KMT2B, KMT2C, KMT2D, KRAS, LAMP1,*

*LATS1, LATS2, LCK, LGI1, LIFR, LIG4, LMO1, LPP, LRP1B, LRRK2, LTK,*

*LYL1, LYN, LZTR1, MAF, MAFB, MAGEA1, MAGI2, MALT1, MAML1, MAP2K1,*

*MAP2K2, MAP2K4, MAP3K1, MAP3K6, MAPK1, MAPK8, MAPK8IP1, MAX,*

*MBD1, MC1R, MCL1, MDM2, MDM4, MECOM, MED12, MEF2B, MEN1, MET,*

*MGA, MITF, MLH1, MLH3, MLLT10, MLLT3, MMP2, MN1, MOB1A, MOB1B,*

*MPL, MPO, MRE11A, MSH2, MSH3, MSH6, MSR1, MTHFR, MTOR, MTR,*

*MTRR, MUC1, MUTYH, MXI1, MYB, MYC, MYCL, MYCN, MYD88, MYH11,*

*MYH9, NAT1, NAT2, NBN, NCOA1, NCOA2, NCOA3, NCOR1, NF1, NF2,*

*NFE2L2, NFKB1, NFKB2, NFKBIA, NIN, NKX2-1, NKX3-1, NOTCH1, NOTCH2,*

*NOTCH3, NPM1, NRAS, NSD1, NTRK1, NTRK2, NTRK3, NUMA1, NUP93,*

*NUP98, PAK3, PALB2, PALLD, PARK2, PAX3, PAX5, PAX7, PBRM1, PBX1,*

*PCBP1, PDCD1LG2, PDGFB, PDGFRA, PDGFRB, PDK1, PER1, PHF6,*

*PHOX2B, PIK3C2B, PIK3CA, PIK3CB, PIK3CD, PIK3CG, PIK3R1, PIK3R2,*

*PIM1, PKHD1, PLCG1, PLCG2, PML, PMS1, PMS2, POLD1, POLE, POLH,*

*POLQ, POT1, POU2AF1, POU2F2, POU5F1, PPM1D, PPP2R1A, PRDM1,*

*PRDM16, PREX2, PRF1, PRKACA, PRKAR1A, PRKCI, PRKD1, PRKDC,*

*PRSS1, PRSS8, PRX, PSIP1, PSPH, PTCH1, PTEN, PTGS2, PTPN11,*

*PTPRC, PTPRD, PTPRT, QKI, RAC1, RAD21, RAD50, RAD51, RAD51B,*

*RAD51C, RAD51D, RAF1, RALGDS, RARA, RASA1, RASAL1, RB1, RBM10,*

*RBM15, RECQL, RECQL4, REL, RET, RHEB, RHOA, RHOH, RICTOR, RINT1,*

*RNASEL, RNF2, RNF43, ROS1, RPL22, RPL5, RPTOR, RRM1, RUNX1,*

*RUNX1T1, RXRA, RYR1, SACS, SAV1, SBDS, SDHA, SDHAF2, SDHB,*

*SDHC, SDHD, SELP, SEMA4A, SEPT9, SETBP1, SETD2, SETDB1, SF3B1,*

*SGK1, SH2D1A, SIN3A, SKP2, SLC15A2, SLC1A3, SLC22A1, SLC22A2,*

*SLC22A6, SLC26A3, SLCO1B1, SLCO1B3, SLIT2, SLX4, SMAD2, SMAD3,*

*SMAD4, SMARCA4, SMARCB1, SMARCE1, SMC1A, SMC3, SMO, SMUG1,*

*SNCAIP, SOCS1, SOS1, SOX10, SOX11, SOX17, SOX2, SOX9, SPEN,*

*SPINK1, SPOP, SPRED1, SPTA1, SRC, SRD5A2, SRSF2, SSX1, STAG2,*

*STAT3, STAT4, STAT5B, STK11, STK3, STK4, SUFU, SULT1A1, SUZ12, SYK,*

*TAF1, TAF15, TAL1, TAP1, TBL1XR1, TBX3, TCF3, TCF7L1, TCF7L2, TCL1A,*

*TERC, TERF2IP, TERT, TET1, TET2, TFE3, TGFBR2, THBS1, TIMP3, TLR4,*

*TLX1, TLX3, TMEM127, TMPRSS2, TNF, TNFAIP3, TNFRSF14, TNK2, TOP1,*

*TOP2A, TP53, TP53BP1, TPMT, TPX2, TRAF3, TRAF7, TRIM24, TRRAP,*

*TSC1, TSC2, TSHR, TYMS, U2AF1, UBR5, UGT1A1, UGT2B15, UGT2B17,*

*UGT2B7, UIMC1, USP9X, VEGFA, VHL, VKORC1, WASF3, WHSC1, WISP3,*

*WRN, WT1, WWTR1, XPA, XPC, XPO1, XRCC1, XRCC2, YAP1, ZBTB2,*

*ZFHX3, ZNF217, ZNF703*

**2. Primer and Probes for TaqMan-Assays**

|  | **Forward Primer** | **Reverse Primer** |
| --- | --- | --- |
| **ETV6** | CAGAACAGAACAAACATGACCTATGAGA | CCTGGCTCCTTCCTGATAATGTTTA |
| **TP53** | CTTTCTTGCGGAGATTCTCTTCCT | GCTTTGAGGTGCGTGTTTGTG |
| **BCL10** | GCAATAAAGTGTCATTGTCGTGAAACA | GGAACTTGTGCAAACTCTAGTGAGA |
|  | **Probe VIC** | **Probe FAM** |
| **ETV6** | AGCCCTGCGCCACTA | AGCCCTGCACCACTA |
| **TP53** | AGAGACCGGCGCACAG | AGAGACCAGCGCACAG |
| **BCL10** | CTTCCCTTAAGATCACG | CTTCCCTTAACATCACG |

**3. digital droplet PCR (dPCR)**

In the ddPCR workflow templates, PCR and assay reagents are dispensed in 15000-20000 separate reaction chambers through generation of nanoliter sized oil droplets. After thermocycling, every droplet is read out for fluorescence. This results in a binary signal for each yellow (VIC®) and green (6-fluorescein, FAM™) channel, which allows to distinguish four populations of droplets: VIC-/FAM- as empty droplets, VIC+/FAM- as wildtype positive droplets, VIC-/FAM+ as mutant positive droplets and VIC+/FAM+ as double positive droplets. Because the distribution pattern of the templates follows poisson mathematics, an appropriate statistical correction allows precise measurement of absolute mutant and wildtype template concentrations from these data. To perform ddPCR we followed BioRad’s standard protocol (BioRad, Hercules, USA). Up front, the DNA amount of every sample (blood derived DNA, tumor tissue derived DNA and cfDNA) was estimated with fluorescence quantification (Qubit dsDNA HS Assay; Thermo Fisher, Waltham, MA, USA) to make sure not to exceed an input of 66ng DNA per ddPCR reaction well, as the manufacturer recommends. In addition, DNA fragment analysis was performed to rule out contamination with cellular DNA (High Sensitivity DNA Kit, Bioanalyzer 2100, Agilent, Santa Clara, USA). Each custom TaqMan assay was tested for its optimum annealing temperature for thermocycling to achieve maximal signal discrimination between droplet clusters, using a thermal gradient protocol for the C1000 thermocycler (BioRad, Hercules, CA, USA) (Fig. S1, S2). This lowers the annealing temperature in a 96-well PCR plate column from 66°C to 56°C in sequential steps. The parameters for thermocycling were as follows: 95°C/10min for initial enzyme activation, 94°C/30s denaturation (42x), variable annealing temperature/60s (42x) and a final enzyme deactivation step at 98°C/10min. For each assay we filled two columns with concordant leukocyte derived DNA as a negative control and tumor tissue DNA as a positive control. The highest temperature, just before which top-level discrimination between droplet clusters could be achieved, was selected to avoid amplification of unspecific targets by maintaining good amplification efficiency.

Because the volume was high and impossible to cover with one single reaction, the main samples were subdivided into several reaction wells until the whole sample volume was spent. If necessary, a small amount of water was added to fit reaction size. In each run corresponding tumor control, blood control and no template control were included. Data analysis was carried out with the program QuantaSoft (BioRad, Hercules, CA, USA). Fluorescence thresholds were set in respect to the controls’ results. *Aliquot results were pooled in silico and handled like a single sample.*

To accommodate for a small fraction of false double positives we determined the false double positive rate (R_fp_) for the VIC+ population as the double positives (N^FAM+VIC+^) in the negative control (C^neg^), with the assumption of the same threshold as in the sample, divided through the total amount of wildtype-only positives (N^FAM-VIC+^).

$$R_{fp}=\frac{N^{FAM+VIC+}}{N^{FAM-VIC+}}$$

To compensate this, admittedly small, error, we corrected the numbers of droplets that went into analysis as follows:

$${N_{c}}^{FAM+VIC+}=N^{FAM+VIC+}-N^{FAM-VIC+}*R_{fp}$$

and

$${N_{c}}^{FAM-VIC+}=N^{FAM-VIC+}+N^{FAM+VIC+}*R_{fp}$$

With the corrected numbers of droplets, we performed the same poisson corrected MAF calculation like the manufacturer’s software (QuantaSoft; BioRad, Hercules, CA, USA):

$$MAF=\frac{-ln(\frac{N^{all}-\left( N^{FAM+VIC-}+N^{FAM+VIC+} \right)}{N^{all}})}{-\ln\left( \frac{{N^{all}-(N}^{FAM-VIC+}+N^{FAM+VIC+})}{N^{all}} \right)-ln(\frac{N^{all}-(N^{FAM+VIC-}+N^{FAM+VIC+})}{N^{all}})}$$

To calculate absolute mutant template concentration in the sample (MTC; copies/μl), we used the following term, based on a mean droplet volume of 850 picolitres and sample input *I* (μl) per reaction:

$$MTC=\frac{-ln(\frac{N^{all}-\left( N^{FAM+VIC-}+N^{FAM+VIC+} \right)}{N^{all}})}{0,00085}*\frac{20}{I}$$

**4. Specific Mutation Pattern of tumors**

| Nr. | chromosome | gene | position | mutation | amino acid change | nucleotide change |
| --- | --- | --- | --- | --- | --- | --- |
| 1 | chr1 | DPYD | 97770854 | missense | p.V754M | c.2260G>A |
| 1 | chr3 | MYD88 | 38182337 | stop_gained | p.Q153* | c.457C>T |
| 1 | chr3 | LTF | 46480843 | missense | p.V574M | c.1720G>A |
| 1 | chr4 | LPHN3 | 62936047 | synonymous | p.R1277R | c.3831C>A |
| 1 | chr4 | EPHA5 | 66356282 | missense | p.C405W | c.1215T>G |
| 1 | chr4 | FAT1 | 187530338 | missense | p.T3402M | c.10205C>T |
| 1 | chr6 | IRF4 | 393269 | complex | p.G39G | c.117G>A |
| 1 | chr6 | PIM1 | 37138168 | complex |  | c.-184G>A |
| 1 | chr6 | PRDM1 | 106536324 | splice_region | p.E97E | c.291G>A |
| 1 | chr6 | SYNE1 | 152644681 | synonymous | p.A5283A | c.15849C>T |
| 1 | chr8 | CSMD3 | 113421279 | intronic |  | c.5276-18C>T |
| 1 | chr8 | MYC | 128748842 | initiator_codon | p.L1L | c.3G>C |
| 1 | chr10 | POLR3A | 79770289 | missense | p.N528H | c.1582A>C |
| 1 | chr10 | BLNK | 98031147 | synonymous | p.K3K | c.9G>A |
| 1 | chr12 | ETV6 | 11803089 | essential_splice_site | p.I10fs | c.29_33+9delTTAAGGTAAAAATC |
| 1 | chr12 | ETV6 | 11803095 | essential_splice_site |  | c.33+1G>A |
| 1 | chr12 | EP400 | 132539661 | missense | p.V2598M | c.7792G>A |
| 1 | chr15 | FES | 91433185 | missense | p.R290W | c.868C>T |
| 1 | chr16 | SOCS1 | 11349078 | synonymous | p.V86V | c.258G>A |
| 1 | chr17 | STAT3 | 40474429 | missense | p.K658E | c.1972A>G |
| 1 | chr17 | CD79B | 62006644 | komplex | p.V212A | c.635T>C |
| 1 | chr17 | SEPT9 | 75369571 | intronic |  | c.23-28570C>T |
| 1 | chr18 | BCL2 | 60985319 | komplex | p.G194V | c.581G>T |
| 1 | chrX | TFE3 | 48900687 | synonymous | p.V22V | c.66G>A |
| 2 | chr12 | MDM2 | 69202261 | kozak_sequence | p.V2M | c.4G>A |
| 3 | chr3 | MYD88 | 38182641 | stop_lost | p.*160R | c.478T>C |
| 3 | chr3 | SETD2 | 47155341 | intronic |  | c.4715+25C>T |
| 3 | chr3 | PBRM1 | 52637660 | frameshift | p.L886fs | c.2655delT |
| 3 | chr3 | PBRM1 | 52696229 | stop_gained | p.E150* | c.448G>T |
| 3 | chr3 | MAGI1 | 66023892 | missense | p.T31M | c.92C>T |
| 3 | chr4 | KIT | 55569883 | splice_region |  | c.757-7C>T |
| 3 | chr4 | LPHN3 | 62936417 | missense | p.E1401K | c.4201G>A |
| 3 | chr5 | MTRR | 7885930 | synonymous | p.C340C | c.1020C>T |
| 3 | chr6 | PIM1 | 37138325 | 5_prime_UTR |  | c.-27C>T |
| 3 | chr6 | PIM1 | 37138326 | 5_prime_UTR |  | c.-26C>T |
| 3 | chr6 | PIM1 | 37138369 | synonymous | p.I6I | c.18C>T |
| 3 | chr6 | PIM1 | 37138420 | synonymous | p.T23T | c.69C>T |
| 3 | chr6 | PIM1 | 37138549 | splice_region | p.G28D | c.83G>A |
| 3 | chr6 | PIM1 | 37138791 | missense | p.S75F | c.224C>T |
| 3 | chr6 | PRDM1 | 106536324 | missense | p.E97D | c.291G>C |
| 3 | chr11 | ATM | 108121582 | missense | p.L464V | c.1390T>G |
| 3 | chr12 | KMT2D | 49447295 | stop_gained | p.W268* | c.803G>A |
| 3 | chr14 | FANCM | 45645802 | missense | p.P1282Q | c.3845C>A |
| 3 | chr15 | IDH2 | 90631755 | intronic |  | c.535-21G>A |
| 3 | chr17 | TP53 | 7577093 | missense | p.R282Q | c.845G>A |
| 3 | chr17 | RNF213 | 78310200 | intronic |  | c.4527+22A>C |
| 3 | chr20 | ASXL1 | 31016134 | inframe | p.L127_D128del | c.381_383delTGA |
| 3 | chr20 | TOP1 | 39708708 | intronic |  | c.336-17_336-16insT |
| 3 | chr22 | BCR | 23523670 | missense | p.P175S | c.523C>T |
| 3 | chr22 | MN1 | 28195197 | synonymous | p.F445F | c.1335C>T |
| 4 | chr12 | CDKN1B | 12870751 | 5_prime_UTR |  | c.-23G>A |
| 4 | chr18 | BCL2 | 60985917 | 5_prime_UTR |  | c.-18G>A |
| 4 | chr13 | FOXO1 | 41239873 | frameshift | p.K151Sfs*174 | c.452_476del |
| 4 | chr14 | NIN | 51237139 | frameshift | p.N467Kfs*20 | c.1400dupA |
| 4 | chr3 | MYD88 | 38181430 | inframe | p.S150del | c.447_449delCAG |
| 4 | chr12 | SLCO1B1 | 21294601 | intronic |  | c.84+9A>G |
| 4 | chr1 | BCL10 | 85733335 | missense | p.R226T | c.677G>C |
| 4 | chr2 | LRP1B | 141027835 | missense | p.P4408L | c.13223C>T |
| 4 | chr2 | FN1 | 216262403 | missense | p.P1173S | c.3517C>T |
| 4 | chr6 | CARD11 | 2984163 | missense | p.G123C | c.367G>T |
| 4 | chr9 | PAX5 | 37002668 | missense | p.T194I | c.581C>T |
| 4 | chr12 | ETV6 | 11803072 | missense | p.T4I | c.11C>T |
| 4 | chr12 | ETV6 | 11803094 | missense | p.K11N | c.33G>C |
| 4 | chr14 | TCL1A | 96178637 | missense | p.P73S | c.217C>T |
| 4 | chr16 | SOCS1 | 11349290 | missense | p.A16T | c.46G>A |
| 4 | chr17 | CD79B | 62007640 | missense | p.S75I | c.224G>T |
| 4 | chr20 | PTPRT | 40727174 | missense | p.A1245T | c.3733G>A |
| 4 | chr3 | FOXP1 | 71179644 | splice_region |  | c.186+5G>C |
| 4 | chr14 | HSP90AA1 | 102552464 | splice_region |  | c.163-3C>T |
| 4 | chr6 | DST | 56328542 | stop_gained | p.E4947* | c.14839G>T |
| 4 | chr12 | KMT2D | 49427369 | stop_gained | p.R3707* | c.11119C>T |
| 4 | chr1 | MCL1 | 150551578 | synonymous | p.= | c.429G>A |
| 4 | chr3 | FOXP1 | 71179712 | synonymous | p.= | c.123C>T |
| 4 | chr17 | PPM1D | 58678156 | synonymous | p.= | c.381G>A |
| 5 | chr2 | LRP1B | 142888306 | 5_prime_UTR |  | c.-8C>T |
| 5 | chr2 | STAT4 | 191931144 | intronic |  | c.630+12A>C |
| 5 | chr2 | PCBP1 | 70315621 | missense | p.A249V | c.746C>T |
| 5 | chr2 | LRP1B | 141528552 | missense | p.N1842H | c.5524A>C |
| 5 | chr2 | ERBB4 | 213403185 | missense | p.D24N | c.70G>A |
| 5 | chr3 | EPHA3 | 89259698 | intronic |  | c.814+28G>T |
| 5 | chr3 | FOXP1 | 71179663 | missense | p.L58V | c.172C>G |
| 5 | chr3 | TBL1XR1 | 176756200 | missense | p.W316C | c.948G>T |
| 5 | chr3 | TBL1XR1 | 176765153 | missense | p.G267C | c.799G>T |
| 5 | chr3 | BCL6 | 187443373 | missense | p.R585W | c.1753C>T |
| 5 | chr3 | FOXP1 | 71179644 | splice_region |  | c.186+5G>A |
| 5 | chr3 | MYD88 | 38182641 | stop_lost | p.*160R | c.478T>C |
| 5 | chr6 | HLA-B | 31323943 | essential_splice_site |  | c.619+1G>A |
| 5 | chr6 | IRF4 | 393270 | missense | p.L40V | c.118C>G |
| 5 | chr6 | PIM1 | 37138583 | missense | p.Q39H | c.117G>T |
| 5 | chr6 | HSP90AB1 | 44221300 | missense | p.D714N | c.2140G>A |
| 5 | chr6 | PIM1 | 37138423 | synonymous | p.= | c.72G>A |
| 5 | chr6 | HLA-C | 31238849 | essential_splice_site |  | c.619+1G>A |
| 5 | chr6 | PIM1 | 37138733 | intronic |  | c.190-24C>G |
| 5 | chr6 | PIM1 | 37138738 | intronic |  | c.190-19G>A |
| 5 | chr6 | PIM1 | 37138820 | intronic |  | c.240+13C>T |
| 5 | chr6 | PIM1 | 37138919 | missense | p.P87A | c.259C>G |
| 5 | chr6 | PIM1 | 37138951 | missense | p.S97R | c.291C>G |
| 5 | chr6 | PIM1 | 37139072 | missense | p.A138T | c.412G>A |
| 5 | chr6 | PIM1 | 37138798 | stop_gained | p.W77* | c.231G>A |
| 5 | chr6 | PIM1 | 37138935 | stop_gained | p.L93* | c.277_286del |
| 5 | chr6 | PIM1 | 37138631 | synonymous | p.= | c.165C>T |
| 5 | chr6 | PIM1 | 37139149 | synonymous | p.= | c.489G>A |
| 5 | chr6 | PIM1 | 37139209 | synonymous | p.= | c.549G>A |
| 5 | chr8 | MYC | 128749712 | intronic |  | c.31-782C>T |
| 5 | chr8 | RECQL4 | 145738585 | intronic |  | c.2463+16C>A |
| 5 | chr8 | MYC | 128746620 | upstream_gene |  |  |
| 5 | chr9 | MLLT3 | 20448290 | intronic |  | c.277-27dupT |
| 5 | chr10 | ARID5B | 63661942 | missense | p.H16Y | c.46C>T |
| 5 | chr11 | ETS1 | 128391855 | missense | p.T12I | c.35C>T |
| 5 | chr11 | ETS1 | 128391858 | inframe | p.P9_T10del | c.26_31delCGACTC |
| 5 | chr12 | CCND2 | 4383343 | missense | p.C46Y | c.137G>A |
| 5 | chr12 | ETV6 | 12038903 | missense | p.R399H | c.1196G>A |
| 5 | chr12 | KMT2D | 49435277 | synonymous | p.= | c.6276C>T |
| 5 | chr16 | CREBBP | 3832786 | missense | p.P491L | c.1472C>T |
| 5 | chr18 | BCL2 | 60985916 | 5_prime_UTR |  | c.-17C>T |
| 5 | chr19 | KEAP1 | 10597383 | missense | p.A607V | c.1820C>T |
| 5 | chr21 | ITGB2 | 46323431 | synonymous | p.= | c.348C>T |
| 5 | chrX | BTK | 100630298 | 5_prime_UTR |  | c.-26C>G |

**5. BioAnalyzer – cfDNA**

As DNA is normally wrapped around histone proteins (146 base pairs (bp) are wrapped around the octameric core particle) and connected with linker fragments of varying size, the length is reflected by a Gaussian distribution around ca. 165 bp. The second and the third peak correlate with multiples of 165 bp. Note that no high molecular DNA was extracted, showing excellent yield of selective isolation of cfDNA. The amount of isolated cfDNA is calculated from the area under the curve (AUC). Figure S3 displays a typical image of a fragment size distribution analysis.

**Figure Legend: Supplementary Material**

Figure S1 **ddPCR Assays optimization (blood):** DNA from the respective blood sample was used as input material to determine the optimal PCR annealing temperature for the detection of the wildtype allele (*TP53* .845G) (Chr.17)).

Figure S2 **ddPCR Assays optimization (tumor):** DNA from the respective tumor tissue was used as input material to determine the optimal PCR annealing temperature for the detection of the mutant allele (*TP53* 845A) (Chr.17).

Figure S3 **Fragment Size Distribution:** Typical image of a fragment size distribution analysis of cell free DNA (cfDNA) after isolation from a blood sample.
