# Supplementary figures and images for: Molecular tumor analysis and liquid biopsy: a feasibility investigation analyzing circulating tumor DNA in patients with central nervous system lymphomas
Source: BMC Cancer. 2019 Mar 1;19:192. doi: 10.1186/s12885-019-5394-x (PMC6397454; doi:10.1186/s12885-019-5394-x)

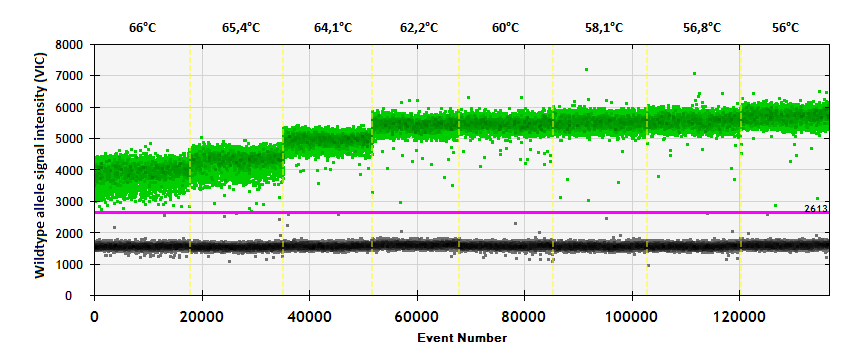

Supplement: Supplementary file 2 — Figure S1. ddPCR Assays optimization (blood). DNA from the respective blood sample was used as input material to determine the optimal PCR annealing temperature for the detection of the wildtype allele (TP53 .845G) (Chr.17)). (TIFF 1210 kb) [file 12885_2019_5394_MOESM2_ESM.tiff]

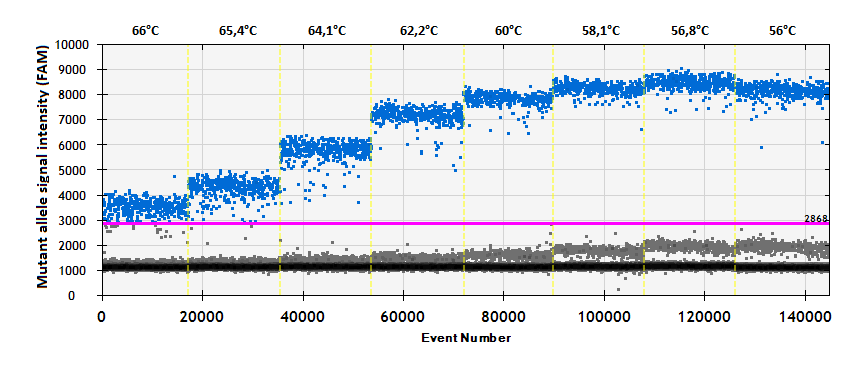

Supplement: Supplementary file 3 — Figure S2. ddPCR Assays optimization (tumor). DNA from the respective tumor tissue was used as input material to determine the optimal PCR annealing temperature for the detection of the mutant allele (TP53 845A) (Chr.17). (TIFF 1239 kb) [file 12885_2019_5394_MOESM3_ESM.tiff]

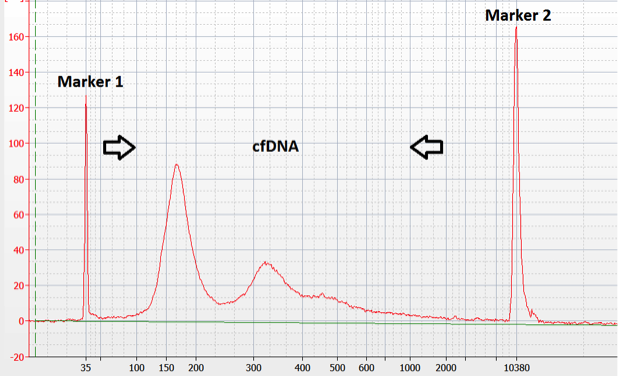

Supplement: Supplementary file 4 — Figure S3. Fragment Size Distribution. Typical image of a fragment size distribution analysis of circulating DNA (cfDNA) after isolation from a blood sample. (TIFF 912 kb) [file 12885_2019_5394_MOESM4_ESM.tiff]
